# Complete Genome Sequence of a New *Firmicutes* Species Isolated from Anaerobic Biomass Hydrolysis

**DOI:** 10.1128/genomeA.00686-17

**Published:** 2017-10-05

**Authors:** Christian Abendroth, Sarah Hahnke, Francisco M. Codoñer, Michael Klocke, Olaf Luschnig, Manuel Porcar

**Affiliations:** a Cavanilles Institute of Biodiversity and Evolutionary Biology, Universitat de València, Paterna, Valencia, Spain; b Institute for Integrative Systems Biology (I2SysBio), Paterna, Valencia, Spain; c Robert Boyle Institut eV, Jena, Germany; d Leibniz Institute for Agricultural Engineering and Bioeconomy (ATB), Bioengineering, Potsdam, Germany; e Lifesequencing SL, Paterna, Valencia, Spain; f Bio H2 Umwelt GmbH, Jena, Germany; g Darwin Bioprospecting Excellence, Paterna, Valencia, Spain

## Abstract

A new *Firmicutes* isolate, strain HV4-6-A5C, was obtained from the hydrolysis stage of a mesophilic and anaerobic two-stage lab-scale leach-bed system for biomethanation of fresh grass. It is assumed that the bacterial isolate contributes to plant biomass degradation. Here, we report a draft annotated genome sequence of this organism.

## GENOME ANNOUNCEMENT

Degrading bacteria, most of them isolated from soil, play relevant roles in the turnover of different types of material, such as petrol (1), pollutants (2), metal (3), and cellulose (4, 5). In the case of plant biomass degradation in biogas reactors, such microorganisms play an important role in making hardly accessible polymeric carbon sources available for other organisms for the production of biogas.

In this study, we present the genome sequence of a new *Firmicutes* isolate, strain HV4-6-A5C, which has a putative role in the microbial metabolic network for plant biomass degradation. This strain was isolated from a lab-scale leach-bed biogas reactor system, which was operated at 37°C with fresh grass as the sole substrate. Isolation was performed on reinforced clostridial agar (Oxoid Ltd.) after the diluted hydrolysate was reincubated with microcrystalline cellulose as the sole carbon source.

We applied a massive genome-sequencing approach using the Illumina NextSeq 500 platform. A Nextera XT library with a mean insert size of 350 nucleotides (nt) was constructed and sequenced with a combination of 150-bp paired-end (PE) reads. A total of 29.2 million PE sequences, with a mean length of 149.85 nt, were obtained. Sequences were filtered by quality, and a total of 29.15 million PE sequences with a *Q* value higher than 20 (mean *Q*, 33.17) were included in the assembly. The sequences were assembled with SPAdes version 3.10.1 (6) using default parameters and a *k*-mer value that provided us with the lowest number of contigs, the longest contig, the largest *N*_50_ value, and the highest percentage of clean sequences. With a *k*-mer value of 77, a total of 106 contigs were obtained. The total size of the genome was approximately 3.3 Mb, with an estimated GC content of 33.43%, a longest contig size of 276,895 bp, and an *N*_50_ value of 113,179 bp.

The assembled genome sequences were annotated using the Prokka version 1.11 annotation pipeline (7), which involved predicting tRNAs, rRNAs, mRNAs, and signal peptides in the sequences using Aragorn, RNAmmer, Prodigal, and SignalP, respectively (8–11).

The genome contains 5,376 elements, of which 5,311 are open reading frames (ORFs) (2,723 canonical and 2,588 noncanonical) and 65 are encoded structural RNAs (sRNAs)—i.e., 5 ORFs for rRNAs and 60 ORFs for tRNAs.

Using BLAST, we compared the contigs with all genome sequences available in the database. According to the PCOP (12) and the AAI (13), the genome can be classified as a species belonging to the genus *Clostridium*. Based on the average nucleotide sequence identity (ANI) (14), the closest related species is *Sporanaerobacter acetigenes*, showing an identity of only 71.13%, which indicates that the novel strain represents a new species within the phylum *Firmicutes*.

### Accession number(s).

The microbial strain reported here has been deposited at DSMZ with the deposit number DSM 104144. The results of the whole-genome project have been deposited at DDBJ/EMBL/GenBank under the accession no. FXVB02000001 to FXVB02000106. The version described here is the first draft version.
